# Implication of Capillary Morphogenesis Gene 2 (CMG2) in the Disease Progression and Peritoneal Metastasis of Pancreatic Cancer

**DOI:** 10.3390/cancers16162893

**Published:** 2024-08-20

**Authors:** Ziqian Fang, Carly Bunston, Yali Xu, Fiona Ruge, Laijian Sui, Ming Liu, Bilal Al-Sarireh, Paul Griffiths, Kate Murphy, Matthew R. Pugh, Chunyi Hao, Wen G. Jiang, Lin Ye

**Affiliations:** 1 Cardiff China Medical Research Collaborative, Division of Cancer & Genetics, School of Medicine, Cardiff University, Cardiff CF14 4XN, UK; fangz7@cardiff.ac.uk (Z.F.); xuy109@cardiff.ac.uk (Y.X.); ruge@cardiff.ac.uk (F.R.); suill@cardiff.ac.uk (L.S.); lium31@cardiff.ac.uk (M.L.); jiangw@cardiff.ac.uk (W.G.J.); 2 Department of Surgery, Morriston Hospital, ABM University Health Board, Swansea SA6 6NL, UK; bilal.al-sarireh@wales.nhs.uk; 3 Department of Pathology, Morriston Hospital, ABM University Health Board, Swansea SA6 6NL, UK; paul.griffiths2@wales.nhs.uk (P.G.); matthew.pugh@wales.nhs.uk (M.R.P.); 4 Key Laboratory of Carcinogenesis and Translational Research, Department of Hepato-Pancreato-Biliary Surgery, Peking University Cancer Hospital & Institute, Beijing 100142, China; haochunyi@bjmu.edu.cn

**Keywords:** capillary morphogenesis gene 2 (CMG2), anthrax toxin receptor 2 (ANTXR2), pancreatic cancer, adhesion, peritoneal metastasis

## Abstract

**Simple Summary:**

Pancreatic cancer remains as one of the most life-threatening cancers with a 5-year overall survival rate less than 6%. As a transmembrane protein, capillary formation gene 2 (CMG2) mediates cell–matrix adhesion and migration. Recent studies have revealed emerging roles of CMG2 in various cancers. This study aimed to evaluate expression of CMG2 in pancreatic cancer and its implication in the disease progression and distant metastasis. Interestingly, the significant upregulation of CMG2 was seen in pancreatic cancer, which was associated with poor survival and distant metastases highlighting the potential of targeting this molecule for the prevention of dissemination of pancreatic cancer cells.

**Abstract:**

Capillary morphogenesis gene 2 (CMG2) mediates cell–matrix interactions to facilitate cell adhesion and migration. CMG2 has been implicated in the disease progression of breast cancer, prostate cancer and gastric cancer. The present study aims to determine the role of CMG2 in the disease progression and peritoneal metastasis of pancreatic cancer. Pancreatic tumour samples were collected from Peking University Cancer Hospital. CMG2 expression was determined using quantitative PCR. After the creation of knockdown and overexpression of CMG2 in pancreatic cancer cells, the effect of CMG2 on several cell functions and adhesion to the peritoneum was examined. Potential pathways regulated by CMG2 were found via proteomics analysis and drug tests. CMG2 was upregulated in pancreatic cancer tissues and associated with a poor prognosis. CMG2 was increased in metastatic lesions and those primary tumours with distant metastases. CMG2 promotes cell–cell, cell–matrix and cell–hyaluronic acid adhesion, which may be mediated by epidermal growth factor receptor (EGFR) and focal adhesion kinase (FAK) pathway activation.

## 1. Introduction

Pancreatic cancer is one of the deadliest cancers. Globally, 495,773 people were diagnosed with pancreatic cancer in 2020, and 466,003 related deaths were reported [1]. Due to a lack of symptoms, the disease is often detected at advanced stages with a significant percentage (80%) of patients presenting with metastases at diagnosis of the disease [2,3]. The prognosis for patients is poor, with a 5-year survival rate of just 6% [4]. Currently, the therapeutic options for patients with pancreatic cancer are limited. The identification of new biomarkers and therapeutic targets is vital in research of pancreatic cancer.

Capillary morphogenesis gene 2 (CMG2), also known as anthrax toxin receptor 2 (ANTXR2), was first identified by Bell et al. who observed that CMG2 was upregulated in endothelial cells undergoing tubule formation in vitro [5]. Both CMG2 and tumour endothelial marker-8 (TEM-8) act as receptors for the intake of anthrax toxin [6,7]. These two proteins share a high homology in their amino acid sequences with 40% identity comprising a von Willebrand factor type A/inserted (vWA/I) domain and a metal ion-dependent adhesion site (MIDAS) motif [6]. The MIDAS motif chelates a divalent cation enabling binding to the protective antigen (PA) subunit of the anthrax toxin, mediating internalisation of the toxin [6]. In addition to binding to PA, the extracellular domain of CMG2 also interacts with the extracellular matrix (ECM) collagen IV, laminin and fibronectin [5].

CMG2 is widely expressed in normal tissues and is associated with tumour-related angiogenesis [8]. Aberrant expression of CMG2 in both breast and prostate cancer has been reported. In breast cancer, the level of CMG2 transcripts in advanced tumours is lower compared with early-stage tumours. It has also been shown that a reduced CMG2 expression level is associated with a shorter overall survival. CMG2 can inhibit the growth of breast cancer cells in vitro and in vivo [9] but has little impact on the proliferation of an AR-negative prostate cancer cell line (PC-3) [10]. However, CMG2 presents an inhibitory effect on the invasiveness of PC-3 cells [10]. Knockdown of CMG2 resulted in a significant decrease in matrix adherence invasion in prostate cancer cells [10]. Our recent study showed that the reduced CMG2 expression in both breast and prostate cancers can be a result of repression mediated by ER and AR [11]. In contrast to the reduced expression in breast and prostate cancers, upregulated expression of CMG2 has been revealed in glioma and gastric cancer, being associated the disease progression [12,13]. In glioma, CMG2 can promote cell adhesion and migration by upregulating YAP [12]. In gastric cancer, CMG2 can promote cancer cell proliferation, invasion, EMT and stemness [13]. CMG2 can promote the formation of laminar pseudopodia and filopodia in glioma cells. Xu et al. found that this mechanism may lie with the CMG2-induced expression of YAP, a critical factor in the Hippo signalling pathway. This pathway is important in regulating the formation of filopodia and pseudopodia in cancer cells [12]. In soft tissue sarcoma, patients with lower CMG2 expression had poorer survival [14]. It has also been found that CMG2 activates the metastasis-related urokinase-type plasminogen activator (u-PA) system in soft tissue sarcoma, further promoting cell migration, invasion and epithelial mesenchymal transition (EMT) [14]. Other than the uPA system, CMG2 is also positively associated with CD26, which is a member of the dipeptidyl peptidase 4 family, and CD26 is also involved in tissue remoulding and putatively in metastasis [14]. Collectively, these studies indicate that CMG2 is involved in the metastatic traits of cancer cells.

In the present study, we aim to determine the role of CMG2 in the disease progression and peritoneal metastasis of pancreatic cancer.

## 2. Materials and Methods

### 2.1. Cell Lines and Cell Culture

Pancreatic cancer cell lines MiaPaCa-2 (CRL-1420), PANC-1 (CRL-1469), ASPC-1 (CRL1682), MET5A (CRL9444) and 293T (CRL-3216) cell lines were purchased from the American Type Culture Collection (ATCC, Middlesex, UK). Both MiaPaCa-2 and PANC1 were cultured in Dulbecco’s modified Eagle’s medium/nutrient F-12 ham (DMEM) (DMEM-F12; Merck, Feltham, UK), which was supplemented with 10% foetal calf serum (FCS) and a 1% cocktail of antibiotics (100×) (Thermo Fisher Scientific, Waltham, MA, USA), with a final concentration of 100 unit/mL penicillin, 100 µg/mL streptomycin and 250 ng/mL amphotericin B. ASPC-1 cells were cultured with RPMI 1640 Medium (RPMI) (Sigma-Aldrich, Dorset, UK), which was supplemented with 10% FCS and antibiotics. MET5A cells were cultured in M199 medium (MEM199, Sigma-Aldrich, Dorset, UK) with 10% FCS and antibiotics. Gefitinib (400 nM) (Selleck chemical, Cambridge, UK), FAK inhibitor 14 (400 nM) (TOCRIS, Abingdon, UK) and the ERK inhibitor FR18024 (200 nM) (Merck, Feltham, UK) were used in the present study.

### 2.2. Pancreatic Tissue Sample Collection

A cohort of pancreatic cancer tissues was obtained from the Peking University Cancer Hospital as part of an institutional collaboration between Cardiff University and Peking University Cancer Hospital. Clinical and pathological categories and the number of patients in each group are shown in Table 1. All protocols and procedures used were approved by the Peking University Cancer Hospital Research Ethics Committee (MTA01062008), and informed consent was obtained from patients, as previously reported [15].

### 2.3. Public Datasets

The Cancer Genome Atlas (TCGA) includes 151 PDAC samples, 9 normal pancreatic tissues, 3 non-invasive tumours, 8 neuroendocrine tumours, 1 acinar ductal adenocarcinoma,1 distant metastatic sample and 9 unconfirmed samples (http://firebrowse.org/, accessed on 31 October 2019).

GSE71729 contains 61 metastatic and 145 primary PDAC tumours, 46 normal pancreatic tissues and 88 normal tissues derived from matched metastatic organs. In addition to the tissue samples, 17 cell lines were also included in the DNA microarrays to determine gene expression [16].

GSE15471 contains 36 pancreatic cancer tumours and paired adjacent pancreatic tissues, with gene expression profiles determined using Affymetrix U133 Plus 2.0 whole-genome chips [17].

Kmplot was used for online survival analysis [18] (https://kmplot.com/analysis/, accessed on 30 April 2024).

### 2.4. Immunohistochemical Staining of CMG2 in Pancreatic Cancer Tissue Microarray

Expression of the CMG2 protein in human pancreatic cancer was evaluated by conducting immunohistochemical staining for CMG2 in a tissue microarray (TMA) (PA2081, Biomax, Rockville, MN, USA). In brief, after retrieving the antigens and blocking using horse serum (1 drop in 5 mL PBS), the TMA was incubated with an anti-CMG2 antibody (ab70499, 1:500, Abcam, Cambridge, UK) followed by detection using a universal biotin-labelled secondary antibody and Vectastain Universal Elite ABC Kit (PK6200, Vector laboratories, Chicago, IL, USA). A negative control was employed to exclude non-specific staining by conducting the staining without the primary antibody. The staining was evaluated and scored by three pathologists (P.G., K.M. and M.P.).

### 2.5. CMG2 Knockdown and Overexpression

CMG2 shRNA vectors were ordered from the Vectorbuilder (Chicago, IL, USA). PANC-1 and ASPC-1 cell lines were transduced with the CMG2 shRNA lentiviral particles (GCTGATTCCAAGGAGCAAGTT) and scramble shRNA control lentiviral particles (CCTAAGGTTAAGTCGCCTCG). The transduced cells were selected with G418 (250–500 µg/mL) for a duration up to two weeks, followed by maintenance using 50 µg/mL of the G418Ribozyme plasmid, CMG2 expression vectors and empty vectors, which were used to establish CMG2 overexpression, knockdown and control cells as previously reported [10].

### 2.6. RNA Extraction, PCR and QPCR

Total RNA was isolated using TRI reagent (Sigma-Aldrich, Dorset, UK). First-strand cDNA synthesis was undertaken using the iScriptTM cDNA Synthesis Kit (Bio-Rad laboratories, Hertfordshire, UK).

PCR was performed using GoTaq Green MasterMix (Promega, Dorset, UK). Products were visualised using a 1% agarose gel stained with SYBR Safe (Invitrogen, Paisley, UK). The PCR was conducted with an initial denaturation for 5 min at 94 °C followed by 30–35 cycles of amplification containing a 20 s denaturation at 94 °C, 30 s annealing at 55 °C and 30 s elongation at 72 °C for 30 s at each cycle, and a 5 min extension at 72 °C at the end.

QPCR for was performed using FAST 2X qPCR MasterMix (Primer Design, Chandle’s Ford, UK). The cycling condition was as following: step 1: initial denaturing: 95 °C for 10 min, step 2: denaturing: 95 °C for 10 s, step 3: annealing: 55 °C for 35 s, step 4: extension: 72 °C for 10 s. Step 2 to step 4 were repeated for 100 cycles, and the fluorescence intensity was recorded at the annealing step. A threshold was automatically generated by the software of the StepOne plus system (Version 2.3).

Details of primers that were used in the present study for conventional polymerase chain reaction (PCR), quantitative real time PCR (QPCR), CMG2 ribozyme synthesis and amplification of the human CMG2-coding sequence are provided in Supplementary Table S1.

### 2.7. Protein Extraction and Western Blotting

The protein samples were extracted with RIPA lysis buffer followed by separation using SDS-PAGE before electric transferring onto a nitrocellulose membrane. Before probing with a primary antibody and the corresponding secondary antibody, the membrane was blocked with 10% skimmed milk. Information for primary and secondary antibodies is provided in Supplementary Table S2. They were all used at a dilution of 1:1000. Protein bands were visualised using the EZ-ECL kit (Sartorius group, Staffordshire, UK). ImageJ (Version 8, https://imagej.nih.gov/ij/, accessed on 5 June 2024, National Institutes of Health (NIH), Bethesda, MD, USA) was used for semi-quantification. Original western blot images are provided in Supplementary Figures S4 and S5.

### 2.8. In Vitro Cell Proliferation Assay

Cells were seeded in a 96-well plate at 3000 cells/well. Cell growth was assessed after 1, 3 and 5 days. CCK8 (cell counting kit 8) (Munich, Germany) was used to determine the cell population by adding 10 μL of CCK8 solution into each well. After an incubation for 2 h at 37 °C, absorbance was determined at a wavelength of 545 nm.

### 2.9. In Vitro Invasion Assay

Trans-well inserts, with an 8 μm pore size, were coated with 50 μg Matrigel and air-dried. Following rehydration, 20,000 cells were added to each insert. After a 72 h incubation, the cells that had migrated through the matrix were fixed and stained with crystal violet. The absorbance of the acetic acid extract of the crystal violet staining was then determined at 595 nm.

### 2.10. In Vitro Migration Assay

A migration assay was performed as previously described [19]. Two hundred thousand pancreatic cancer cells were seeded into a 24-well plate and left to form a confluent monolayer overnight. Cells were wounded using a 200 μL pipette tip. The EVOS FL Auto Cell Imaging System (Life Technologies, Carlsbad, CA, USA) was used to take photos every hour up to 6 h. ImageJ software (Version 8) (NIH, Bethesda, MD, USA) was employed to measure the migration.

For ASPC-1 cells, which are less adhesive, the microcarrier bead assay was used to determine the migration (Sigma-Aldrich, Dorset, UK). Firstly, 5 g of the Cytodex-2 beads was suspended in 75 mL of PBS, and 1 million cells were then added to 1 mL of bead suspension in a universal container, in 10 mL of culture medium. After overnight incubation, cells remaining suspended were washed away using PBS, cells attached to the beads were subsequently resuspended in 1 mL of medium and 100 µL of the cell suspension was seeded per well in a 96-well plate. After a 3 h incubation, the beads and non-migrated cells were washed off. Cells attached to the bottom of the well were then fixed and stained using crystal violet. The absorbance of the crystal violet extract was determined at 595 nm.

### 2.11. Cell–Matrix Adhesion Assay

A Matrigel (5 μg/well)-pre-coated 96-well plate was used to determine cell–matrix adhesion, in which 20,000 cells were seeded in each well. Following a 40 min incubation, non-adherent cells were removed with a wash using PBS buffer. Adhered cells were then fixed and stained using crystal violet. The absorbance of the crystal violet extract was determined at 595 nm.

### 2.12. Adhesion to Mesothelial Cells

MET5A cells were seeded in a 96-well plate with 200 μL of medium, and after overnight culture, a cell monolayer was formed. After staining with 10 μg/mL DiI (Thermo Fisher Scientific, Waltham, MA, USA), 20,000 pancreatic cancer cells were seeded onto the MET5A monolayer. Following an incubation of 40 min, nonadherent cells were washed off using PBS. Adhered cells were then fixed and photographed using the EVOS FL Auto Cell Imaging System (Life Technologies, Carlsbad, CA, USA).

To evaluate the influence of HA on the adhesion of pancreatic cancer cells to the mesothelial cells, 20,000 pre-stained cancer cells were seeded into each well. During the following 40 min incubation, the cells were treated with HA at different concentrations: 100 μg/mL, 50 μg/mL and 25 μg/mL. High-, low- and ultralow-molecular-weight hyaluronic acid (CLR002, CLR001, CLR003, Sigma-Aldrich, Dorset, UK) was used. Meanwhile, a hyaluronic acid inhibitor (AS-62622, Anaspec, Fremont, CA, USA) at a concentration up to 2 μM was also used to verify the involvement of HA in the adhesion of cancer cells to mesothelial cells. The adhered cells were determined following the aforementioned procedure.

### 2.13. Cell Aggregation Assay

Pancreatic cancer cells were suspended in 5 mL of medium at a density of 1 × 10^5^ cells/mL in a 30 mL universal container followed by an incubation of up to 24 h with a continuous agitation. The number of cell clusters were counted every hour during the first 6 h of the incubation and at the end of the 24 h incubation.

### 2.14. Hoechst Staining to Detect Apoptotic Cells

In the aforementioned aggregation test, 200 μL of the suspended cancer cells were collected every hour during the first 6 h and also at the end of the 24 h suspension culture. After staining with 0.1 μg/100 μL Hoechst (Thermo Fisher Scientific, Waltham, MA, USA) for 20 min, apoptotic cells were counted under a fluorescent microscope. Cells with a condensed or lobular-shaped nucleus were counted as apoptotic cells.

### 2.15. Cell Viability Test with CCK8

At the beginning of the suspension culture, 50 µL of CCK8 (cell counting kit 8) reagent was added into each 5 mL suspension cell culture (1 × 10^5^ cell/mL). After a 4-h incubation, the absorbance, representing viable cells, was measured at 450 nm. For MiaPaCa-2^CMG2exp^ cells, PANC-1^CMG2exp^ cells and the control cells, the duration was 4 h. PANC-1 CMG2-knockdown cells and the controls cell were incubated for 3 h before the absorbance was determined. The duration of the suspension culture for ASPC-1 cells was 6 h.

### 2.16. Flow Cytometric Apoptosis Assay

The Annexin V Apoptosis Detection Kit (sc-4252-AK, Santa Cruz Biotechnology, Inc., Dallas, TX, USA) was applied for the apoptosis assay using a flow cytometer (FACS Canto II, BD biosciences, Berkshire, UK). FITC-labelled Annexin-V stains cells in the early or late apoptosis stage. Propidium iodide (PI), a membrane-impermeable red fluorescent dye, was used to stain dead or late-stage apoptotic cells with a leaking membrane. Apoptotic cells were determined using the flow cytometer at a duration of suspension culture as aforementioned in the apoptosis assays using Hoechst. In brief, the harvested suspended cells were adjusted to a density of 1 × 10^6^ cells/mL, and 100 μL of the adjusted cell suspension were added with 2 μL FITC-Annexin V (0.4 μg Annexin V) and 2 μL of the PI solution. For a positive control, 0.01% H_2_O_2_ was added to induce apoptosis. A negative control was also included in which the cells were not stained with neither Annexin V or PI. After staining at 37 °C for 15 min in an incubator, apoptotic cells were determined using FACS Canto II (BD biosciences, Berkshire, UK). The apoptotic population was analysed with FlowJo (https://www.flowjo.com/, Ashland, OR, USA).

### 2.17. Proteomic Analysis Using Mass Spectrometry

Proteins were extracted from MiaPaCa-2^pEF^, MiaPaCa-2^CMG2exp^, ASPC-1^SC^ and ASPC-1^CMG2shRNA^ cell lines, using RIPA buffer supplemented with phosphatase inhibitors (P5726 and P0044, Merck, Feltham, UK). After quantification of the protein concentration, using a Bio-Rad protein quantification kit (Bio-Rad, Hertfordshire, UK), the concentration of the protein samples was adjusted to 2 mg/mL. Triplicates of each sample were prepared, which contained 200 µg of total proteins. The samples were sent to Bristol University for proteomic analysis, using mass spectrometry at Bristol University. The data were processed and analysed using Proteome Discoverer v2.1 software.

### 2.18. Kinexus Protein Array Analysis

RIPA buffer, without SDS, was applied to extract the protein from PANC-1^pEF^ and PANC-1^CMG2rib^ cell lines. After protein quantification, using a Bio-Rad protein quantification kit (Bio-Rad, Hertfordshire, UK), the concentration was adjusted to 3 mg/mL. Subsequently, 200 μg of protein was used for the protein array, and the KAM-880 antibody microarray kit was applied (Kinexus Bioinformatics Corporation, Vancouver, BC, Canada).

### 2.19. Immunoprecipitation (IP)

Cells were cultured in a 75 cm^2^ flask. After the proteins were extracted and quantified, 2 μg of primary antibody (listed in table) was added to 500 μg of the protein sample. After incubation at room temperature for 2 h, Protein G-, A- or G/A-coupled agarose beads were added (50 μL of beads for 500 μg of protein). After another hour incubation at room temperature, samples were centrifuged at 8000 rpm at 4 °C for 10 min. The pellet included immunoprecipitants being referred as an IP sample, while the supernatant was referred to as a non-IP sample. The original total cell lysate was also prepared as a control.

### 2.20. Statistical Analysis

For a comparison between two groups, the Mann–Whitney test was applied for non-normally distributed data, while a *t*-test was used for normally distributed data. One way ANOVA was employed for statistical analyses of multiple group data. Correlations were determined using a Spearman test. Kaplan–Meier survival analysis was conducted using the KMPlot analysis (www.KMplot.com, accessed on 30 April 2024) [20]. All the statistical analyses were performed using SPSS software (version 26, SPSS, Chicago, IL, USA). Results were considered as statistically significant when *p* < 0.05.

## 3. Results

### 3.1. Upregulated CMG2 in Pancreatic Cancer and Disease Progression

**Figure 1:**
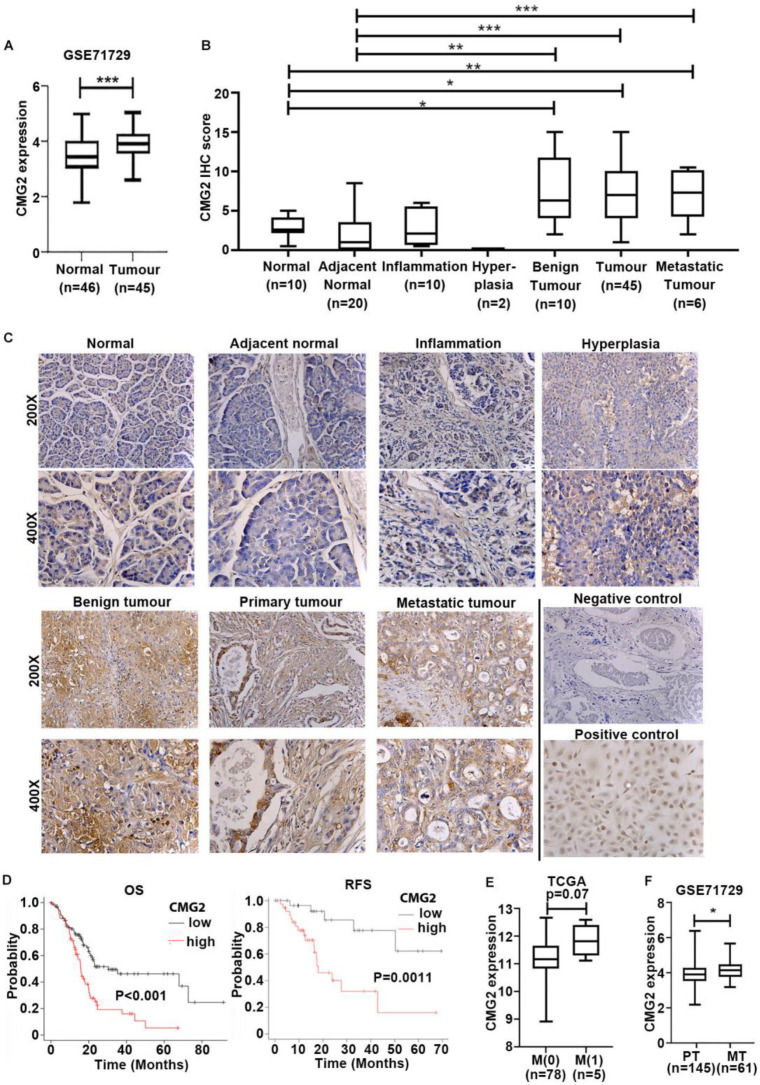
Increased CMG2 expression in pancreatic cancer was associated with disease progression and poor prognosis. (**A**) Expression of CMG2 in pancreatic tumours was analysed in comparison with adjacent normal pancreatic tissues in the public cohort GSE71729 [16] (**B**) Shown are the IHC scores of CMG2 staining in normal pancreas tissues, adjacent normal pancreatic tissues, inflammation tissues, hyperplasia tissues, benign tumour tissues and in primary tumours on the tissue microarray (PA2081). (**C**) Shown are representative images that were reduced from photos taken at magnifications of 200× and 400×. The negative control was staining performed with the secondary antibody only. CMG2-overexpressing HECV cells were included as a positive control for the staining. (**D**) The association between CMG2 expression and patients’ survival was analysed using the KMplot online platform (www.KMplot.com, accessed on 40 April 2024) [20], including overall survival (OS) and relapse-free survival (RFS). (**E**) Shown are CMG2 transcript levels in primary tumours according to the status of distant metastasis in TCGA cohort. (**F**) The expression of CMG2 in metastatic tumours (MT) (*n* = 61) was compared with the primary tumours (*n* = 162) in the GSE71729 cohort. *** *p* < 0.001, ** *p* < 0.01, * *p* < 0.05.

The quantitative analysis of CMG2 transcripts in the Beijing pancreatic cancer cohort revealed the increased expression of CMG2 in pancreatic cancers (*n* = 153), *p* = 0.002, compared with adjacent normal pancreatic tissues (*n* = 174) (Table 1). The increased expression of CMG2 in pancreatic cancer was also observed in the GSE71729 cohort comprising 45 primary tumours and 46 adjacent normal pancreas tissues (Figure 1A). CMG2 protein expression in pancreatic cancer was further examined in the pancreatic cancer TMA (PA2081). Increased CMG2 protein expression was seen in pancreatic cancers (*n* = 45) in comparison with the normal pancreas (n = 10) and adjacent normal pancreatic tissues (*n* = 20) (Figure 1B,C). Enhanced staining of CMG2 was also observed in benign islet tumours compared with normal pancreases (Figure 1B,C).

The association between CMG2 and pancreatic cancer prognosis was analysed using an online platform for Kaplan–Meier survival analysis [20]. Patients with a high level of CMG2 transcripts have a significantly shorter overall survival (OS) (*p* < 0.001) compared with those who had a lower expression level of CMG2. Furthermore, higher CMG2 expression was also associated with a shorter relapse-free survival (RFS) (Figure 1D).

A trend of increasing expression of CMG2 was seen in the primary tumours that presented with distant metastases (M1) at the diagnosis of the disease although this did not reach a statistically significant level, *p* = 0.07, when compared with its expression in primary tumours without distant metastasis (M0) (Figure 1E). Furthermore, the transcript levels of CMG2 were significantly higher in metastatic tumours (*n* = 61) from the pancreatic cancers compared with its expression in the primary tumours (*n* = 162) (Figure 1F). However, such a trend was not seen in those primary tumours from patients who developed distant metastases during the follow-up period (Table 1).

### 3.2. Influence of CMG2 on Cellular Functions of Pancreatic Cancer Cells

**Figure 2:**
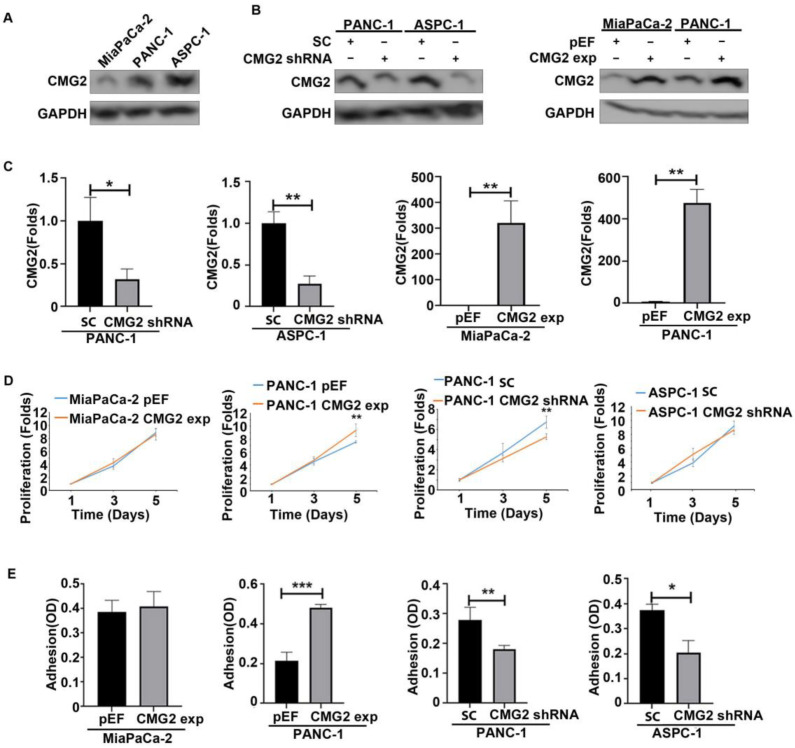
CMG2 regulated pancreatic cancer cell growth and adhesion. (**A**) CMG2 protein expression in MiaPaCa-2, ASPC-1 and PANC-1 cell lines was determined using Western blotting. The knockdown and overexpression were successfully established, which were verified using Western blotting (**B**) and quantitative PCR (**C**), respectively. (**D**) The influence of CMG2 on the proliferation of MiaPaCa-2, PANC-1 and ASPC-1 was determined using CCK8. Six replicates were examined for each cell line in an experiment. (**E**) Adhesion to an artificial basement membrane (Matrigel) was determined in the CMG2-overexpressing MiaPaCa-2 and PANC-1 cell lines and CMG2-knockdown cells of PANC-1 and ASPC-1. Three independent experiments were conducted. *** *p* < 0.001, ** *p* < 0.01, * *p* < 0.05.

CMG2 was highly expressed in ASPC-1 cells, with moderate expression in PANC-1 cells. A lower expression of CMG2 was shown in MiaPaCa-2 cells (Figure 2A). To investigate the impact of CMG2 on the cellular functions of pancreatic cancer cells, lentiviral CMG2 shRNA was employed to knockdown CMG2 expression in ASPC-1 and PANC-1 cells, while the overexpression of CMG2 was established in MiaPaCa-2 and PANC-1 cell lines using the plasmid vectors. CMG2 knockdown and overexpression were verified using Western blotting and quantitative PCR (Figure 2B,C).

Following the verification of the CMG2 knockdown and overexpression, the influence of CMG2 on cell growth and adhesion was determined. There was no obvious change seen in the proliferation of both MiaPaCa-2^CMG2exp^ cells and ASPC-1^CMG2shRNA^ cells, compared with the corresponding control. Interestingly, the knockdown of CMG2 resulted in the inhibition of proliferation of PANC1 cells, while an increase was seen in PANC-1^CMG2exp^ cells (Figure 2D).

The influence on adhesion of pancreatic to an artificial basement membrane matrix (Matrigel) was also determined. A significant decrease in cell adhesion was seen in both ASPC-1 and PANC-1 cells following the knockdown of CMG2, while CMG2 overexpression resulted in the increased adhesion of PANC-1 cells but not the MiaPaCa-2 cells (Figure 2E). However, in the present study, no obvious effect on invasion and migration was observed in the three pancreatic cancer cell lines examined, following the knockdown and overexpression of CMG2 (Supplementary Figure S2A,B).

### 3.3. CMG2 and Adhesion of Pancreatic Cancer Cells to Mesothelial Cells

**Figure 3:**
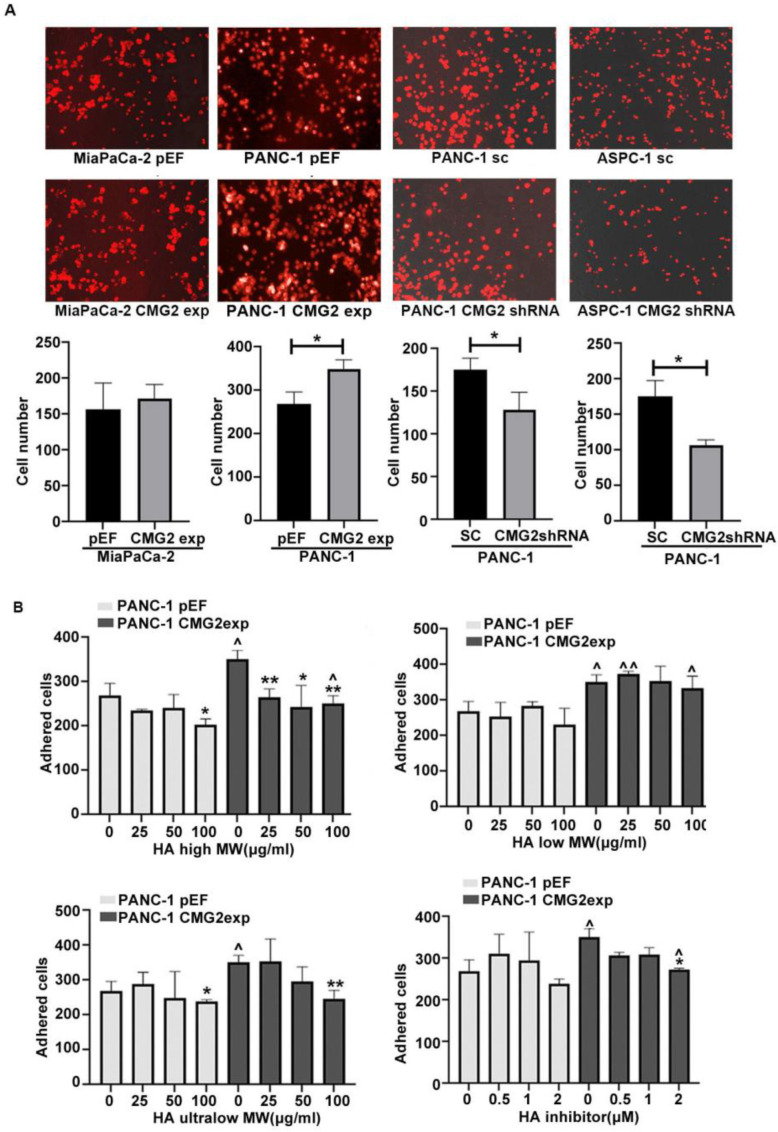
The effect of CMG2 overexpression or knockdown on the peritoneum metastasis of pancreatic cancer. (**A**) Adhesion to mesothelial cells was determined in MiaPaCa-2CMG2exp, PANC-1CMG2shRNA and ASPC-1shRNA against corresponding control groups, respectively. The cells were stained with DiI. Shown are representative photos. (**B**) The PANC-1 cell line with CMG2 overexpression was treated with high-molecular-weight, low-molecular-weight, and ultra-low-molecular-weight hyaluronic acid and a hyaluronic acid inhibitor with different concentrations. Shown are the PANC-1 cell number adhered to the MET5A cell monolayer. * v.s. corresponding untreated controls, ^ v.s. pEF control, ** *p* < 0.01, * *p* < 0.05, ^^ *p* < 0.01 and ^ *p* < 0.05.

To determine whether CMG2 can affect the adhesion to mesothelial cells, pancreatic cancer cell lines with CMG2 overexpression and knockdown were seeded onto a culture plate precoated with a monolayer of MET5A cells. CMG2 knockdown in both ASPC-1 and PANC-1 exhibited the inhibition of adhesion to the mesothelial cells, while CMG2 overexpression enhanced the adhesion of PANC1 cells but had little impact on the adhesion of MiaPaCa-2 cells (Figure 3A).

HA forms a coating film on the lumen surface of the peritoneum, which has been implicated in peritoneal metastasis [21]. To investigate whether the CMG2-enhanced adhesion of cancer cells to mesothelial cells is mediated or partially mediated by HA, PANC-1 cells with CMG2 overexpression were treated with HA of different molecular weights (MW) and an HA inhibitor, respectively. High MW HA inhibited the adhesion of both PANC-1^CMG2exp^ and PANC-1^pEF^ cells to MET5A cells and eliminated the CMG2-overexpression-promoted adhesion of PANC1 cells to the mesothelial cells. However, CMG2-promoted adhesion was not eliminated when a high concentration (100 μg/mL) of high-MW HA was applied. Low-molecular-weight HA of concentrations up to 100 µg/mL did not present an obvious inhibition of the cancer cell-mesothelial cell adhesion in both PANC-1^CMG2exp^ and the control cells. Ultra-low-molecular-weight HA had a similar effect as high-molecular-weight HA, which inhibited the adhesion significantly in PANC-1^CMG2exp^ cells compared with the control cells. The HA inhibitor reduced the adhesion of PANC-1^CMG2exp^ cells, while presented little effect on PANC-1^pEF^ cell adhesion. Except the low-molecule-weight HA, the high-molecule-weight HA, ultralow-molecule-weight and the HA inhibitor elicited the remarkable inhibition of the CMG2-promoted adhesion, though only the ultralow-molecular-weight HA (100 µg/mL) could diminish the CMG2 enhanced adhesion. The high-molecular-weight HA also exhibited inhibition of the adhesion in both PANC-1^CMG2exp^ and the control cells (Figure 3B).

### 3.4. Influence of CMG2 on Survival and Aggregation of Suspended Pancreatic Cancer Cells

**Figure 4:**
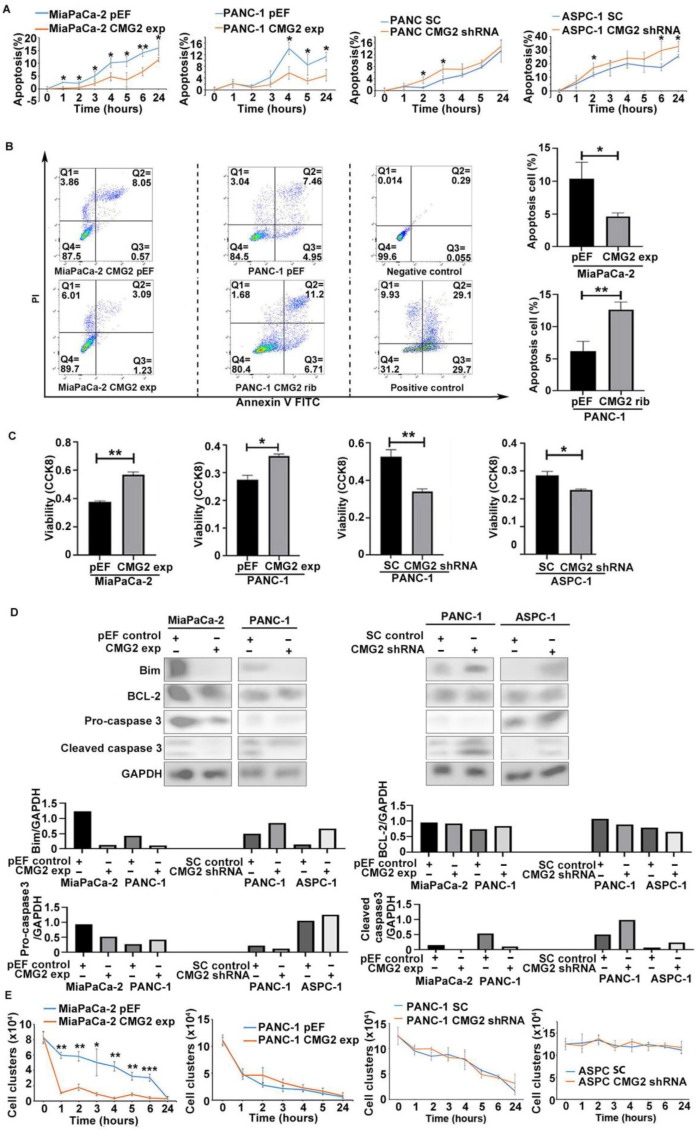
CMG2 and anoikis. (**A**) Hoechst staining was used to determine the apoptotic cells in suspended MiaPaCa-2, PANC-1 and ASPC-1 cells with CMG2 overexpression or knockdown. (**B**) Apoptosis in the suspended MiaPaCa-2 and PANC-1 cells was determined using the flow cytometric apoptosis assay. The apoptotic population included both early apoptotic (Q3) and late apoptotic (Q2) cells. Three independent experiments were conducted. Shown are the representative results from one experiment. (**C**) CCK8 was also used to determine cell viability in MiaPaCa-2, PANC-1 and ASPC-1 cells with CMG2 overexpression or knockdown. (**D**) After a suspension culture of 2.5 h, the expression of Bim and BCL-2 and the activation state of caspase 3 and caspase 8 in MiaPaCa-2, PANC-1 and ASPC-1 cells with CMG2 overexpression and knockdown were shown. Bar graphs show the normalised integrated density of bands against the corresponding GAPDH following the semi-quantification of the bands using Image J (Version 8). (**E**) Cell aggregation was determined in MiaPaCa-2, PANC-1 and ASPC-1 cells. Shown are the numbers of clusters and cells per ml. *** indicates *p* < 0.001, ** indicates *p* < 0.01 and * indicates *p* < 0.05.

Anchorage is essential to maintain the survival of adherent cells. Pancreatic cancer cells encounter a challenge for survival during the dissemination in the peritoneal cavity. We determined the influence of CMG2 on the survival of suspension cancer cells. Upon the loss of anchorage, there was a steady increase in apoptotic populations in both MiaPaCa-2^pEF^ and PANC1^pEF^ cells. Reduced apoptotic populations were seen in both MiaPaCa-2^CMG2exp^ and PANC-1^CMG2exp^ cells over a course of suspension up to 6 h. An increase appeared in the apoptotic cells in both PANC-1 and ASPC-1 cells following the knockdown of CMG2 but to a lesser extent (Figure 4A). A flow cytometric apoptosis assay was also employed to determine the influence CMG2 on anoikis in the pancreatic cancer cells. The PANC-1 cell line with CMG2 knockdown was established using a ribozyme targeting CMG2, which was verified using Western blotting (Supplementary Figure S1A). There was a reduction in the apoptotic population in MiaPaCa-2^CMG2exp^ cells, whilst increased apoptotic cells were seen in the PANC-1 cells following the knockdown of CMG2 (Figure 4B).

Furthermore, a CCK8 assay was used to determine the viability of the suspended cells. In line with the influence of CMG2 on apoptosis, an increased number of viable cells was seen in both MiaPaCa-2^CMG2exp^ and PANC-1^CMG2exp^ cells (Figure 4), whilst reduced viability was evident in the CMG2-knockdown cell lines, including both PANC1 and ASPC-1 (Figure 4C). After a suspension culture for 2.5 h, Bim was reduced in MiaPaCa-2 and PANC-1 cells with CMG2 overexpression, while increased levels were seen in both PANC-1 and ASPC-1 cells with CMG2 knockdown. Caspase 3 is another cell apoptosis protein, of which the activation state was lower in both MiaPaCa-2^CMG2exp^ and PANC-1^CMG2exp^ cells, while elevated activation was seen the cells with CMG2 knockdown (Figure 4D).

During the spreading of cancer cells in the peritoneal cavity, the aggregation and clustering of cancer cells can assist them to survive and escape from the anoikis [22,23]. Clusters formed by the suspended pancreatic cancer cells were determined to evaluate the influence of CMG2 on the aggregation of pancreatic cancer cells upon a loss of anchorage. The number of both cells and cell clusters formed by MiaPaCa-2^CMG2exp^ cells were markedly reduced after 1 h of suspension compared with the pEF control cells, which remained significantly different in comparison with the control until the endpoint of the experiment (24 h). In addition to that, CMG2 did not exhibit a similar impact on the aggregation of PANC-1 cells. There was hardly a change in the aggregation observed in ASPC-1 cells even with the knockdown of CMG2 (Figure 4E).

### 3.5. Regulation of Other Adhesion Molecules in Pancreatic Cancer by CMG2

**Figure 5:**
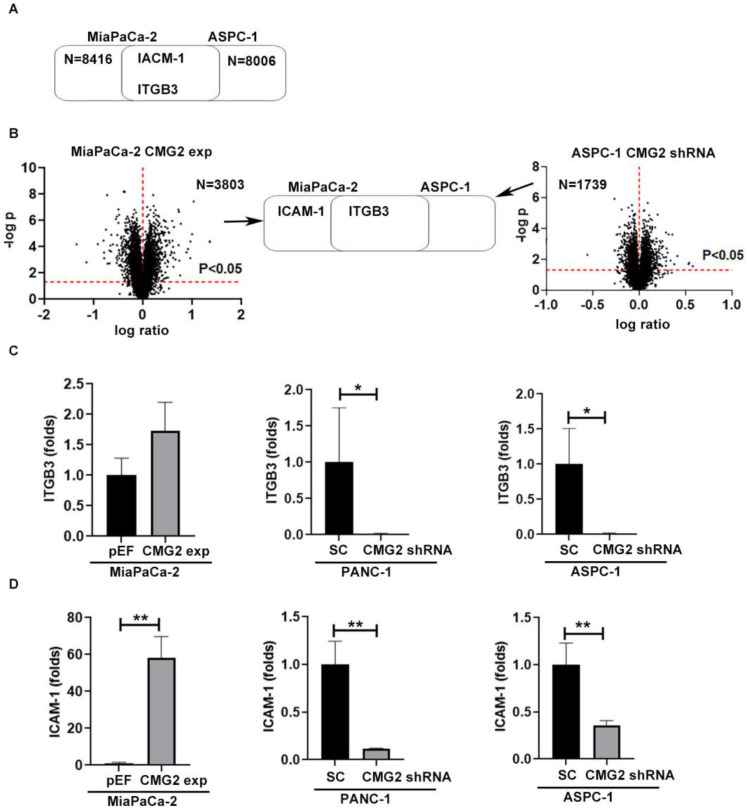
CMG2 regulated cell adhesion molecules. The cell adhesion genes were positively associated with CMG2 at both the transcript level (**A**) and protein level (**B**). Horizontal red dashed lines indicate a level of *p* = 0.05 while vertical dashed lines indicate a change in folds = 0. QPCR results show the transcript changes of ITGB3 (**C**) and ICAM-1 (**D**) after CMG2 overexpression or knockdown in MiaPaCa-2, PANC-1 and ASPC-1 cells. ** *p* < 0.01, * *p* < 0.05.

To further investigate how CMG2 regulates cell adhesion, RNA sequence and proteomics analyses were conducted. As revealed in the RNA sequencing data, there were 8416 genes upregulated in the MiaPaCa-2^CMG2exp^ cell line and 8006 genes downregulated in ASPC-1^CMG2shRNA cells^ in comparison with the respective control (Figure 5A). The proteomic analysis showed that there were 3803 proteins significantly upregulated in the MiaPaCa-2^CMG2exp^ cells, while 1739 proteins were significantly downregulated in ASPC^CMG2shRNA^ cells (Figure 5B). ICAM-1 and ITGB3 were found to be positively associated with CMG2 expression in pancreatic cancer cell lines at both the transcript and protein levels (Figure 5A,B). QPCR was employed to determine the transcript level of the candidate genes. ITGB3 was significantly downregulated in ASPC-1^CMG2shRNA^ cells (Figure 5C). ICAM-1 expression was significantly increased in the MiaPaCa-2^CMG2exp^ cell line, while decreased expression was seen both in PANC-1 and ASPC-1 cell lines following the knockdown of CMG2 (Figure 5D).

### 3.6. CMG2 Upregulated Other Adhesion Molecules through Both EGFR and FAK Pathways

**Figure 6:**
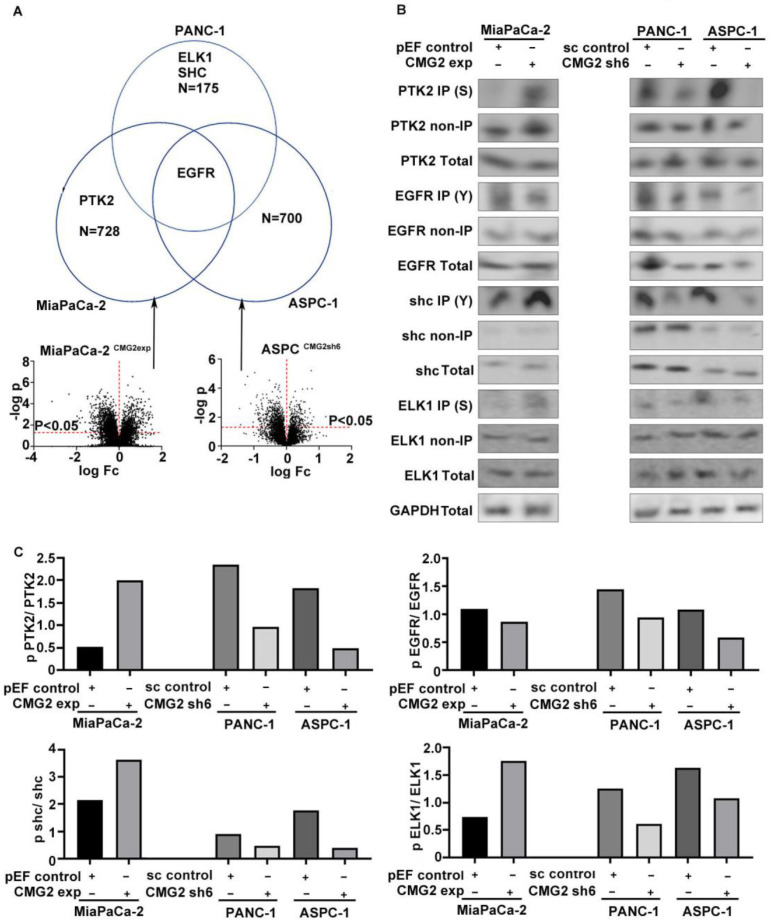
Protein phosphorylation regulated by CMG2. (**A**) The phosphorylation status of the key proteins that enhance cell adhesion was analysed. Shown are the phosphorylation statuses of these proteins that are positively associated with CMG2 in proteomics and Kinexus protein array results. Horizontal red dashed lines indicate a level of P = 0.05 while vertical dashed lines indicate a change in folds = 0. (**B**) The phosphorylation status of candidate proteins was verified in pancreatic cancer cell lines with CMG2 overexpression and knockdown. (**C**) Shown are the semi-quantification result of the phosphorylation status of PTK2, EGFR, shc and ELK1.

According to the proteomic analyses, 728 proteins had an increase of adjusted phosphorylation in the MiaPaCa-2^CMG2exp^ cells, while 700 proteins presented an adjusted decrease in phosphorylation in the ASPC-1^CMG2shRNA^ cells. These phosphorylated proteins were positively correlated with CMG2 expression in both cell lines. In the PANC-1 cells with CMG2 knockdown, the Kinexus protein array revealed the decreased phosphorylation level in 175 proteins. Among those proteins, the phosphorylation state of ELK1 (ETS Transcription Factor ELK1), SHC (Src Homology 2 Domain-Containing Transforming Protein), EGFR (Epithelial growth factor receptor) and PTK2 (Focal adhesion kinase) was positively correlated with CMG2 (Figure 6A), which are involved in EGFR and FAK pathways.

PTK2 with phosphorylated serine, also known as FAK (Focal adhesion kinase), was increased in MiaPaCa-2^CMG2exp^ cells compared with the control, while the phosphorylated PTK2 was decreased in both PANC-1 and ASPC-1 cells, following the knockdown of CMG2 (Figure 6B). EGFR expression was decreased in PANC-1^CMG2shRNA^ and ASPC-1^CMG2shRNA^ cells. The phosphorylation state of EGFR at tyrosine residues appeared to be less in both PANC-1 and ASPC-1 following the knockdown of CMG2. A marked increase in phosphorylated Shc (p-Shc) with tyrosine (Y) phosphorylation was seen in MiaPaCa-2CMG2exp cells, while reduced p-Shc (Y) was seen in both PANC-1^CMG2shRNA^ and ASPC-1^CMG2shRNA^ cell lines (Figure 6B,C). Similar changes were also seen in ELK1 with serine phosphorylation but to a lesser level (Figure 6B,C).

**Figure 7:**
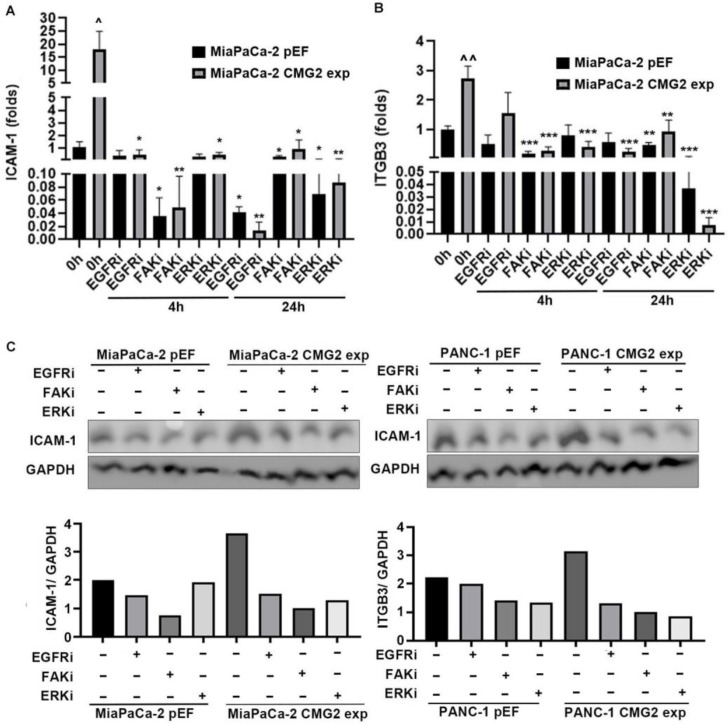
EGFR, ERK and FAK in CMG2-regulated ICAM-1 and ITGB3. QPCR was performed to check the expression of ICAM-1 (**A**) and ITGB3 (**B**) in the MiaPaCa-2 cell line with CMG2 overexpression, which was treated with an EGFR inhibitor (Gefitinib, 400 nM), FAK inhibitor 14 (400 nM) and ERK inhibitor (FR18024, 200 nM). Cell lines were treated with small inhibitors for 4 and 24 h * v.s. untreated control, ^ v.s. pEF corresponding control. (**C**) Shown are ICAM-1 expression at the protein level in MiaPaCa-2 and PANC-1 cell lines with CMG2 overexpression and in their corresponding pEF control cell lines. Cells were treated with an EGFR inhibitor (Gefitinib, 400 nM), FAK inhibitor 14 (400 nM) and ERK inhibitor (FR18024, 200 nM) for 24 h. Bar graphs show the normalised integrated density of bands against the corresponding GAPDH following semi-quantification of the bands using Image J (Version 8). *** *p* < 0.001, ** *p* < 0.01, * *p* < 0.05, ^^ *p* < 0.01, ^ *p* < 0.05.

To examine whether the EGFR pathway and FAK pathway were involved in CMG2-regulated intercellular Adhesion Molecular 1 (ICAM-1) and integrin β3 (ITGB3), small inhibitors targeting EGFR, FAK and ERK were employed. ICAM-1 expression in MiaPaCa-2^CMG2exp^ cells was significantly decreased after a 4 h incubation with Gefitinib (Figure 7A), while ITGB3 expression was decreased significantly after treatment with Gefitinib for 24 h (Figure 7B).

Upregulated ICAM-1 expression in the MiaPaCa-2^CMG2exp^ cell line was decreased significantly after treatment with the ERK inhibitor (Figure 7A,B). Similar changes in ICAM-1 and ITGB3 were also observed in PANC-1 cells that were treated with the inhibitors. (Supplementary Figure S3). ICAM-1 expression in MiaPaCa-2 and PANC-1 treated with small inhibitors for 24 h was further determined using Western blotting. Its expression was decreased in the cells that were treated with the small inhibitors, and the difference between control and CMG2-overexpressing cells was diminished by these inhibitors (Figure 7C).

## 4. Discussion

CMG2 expression was increased significantly in pancreatic cancers compared with adjacent normal tissues in the Beijing cohort. This is supported by findings in the analysis of other pancreatic cancer cohorts and IHC staining of CMG2 in the pancreatic cancer TMA. Increased CMG2 expression was associated with poor survival in pancreatic cancer. CMG2 expression was increased in distant metastases from pancreatic cancer, in comparison with its expression in the primary tumours. In pancreatic cancer, distant metastasis usually leads to a poor prognosis. Even micro-metastasis can also cause a dismal outcome, and these patients cannot benefit from the local treatments [24]. Reduced expression of CMG2 has been previously revealed in both prostate cancer and breast cancer, which are associated with the poor prognosis of those diseases [9,10]. The reduced expression of CMG2 in the two endocrine-related cancers can be a result of repression induced by sexual hormones [11]. In contrast to breast and prostate cancers, the present study revealed the elevated expression of CMG2 in pancreatic cancer, and the increased expression of CMG2 was associated with disease progression and poor prognosis. In line with our findings in pancreatic cancer, increased expression of CMG2 was also observed in gastric cancer [13], suggesting that CMG2 plays a different role in gastrointestinal cancers in comparison with the endocrine-related cancers. CMG2 knockdown markedly impaired the adhesion of both PANC-1 and ASPC-1 cells. In line with this finding, the overexpression of CMG2 enhanced adhesion in PANC-1 cells. This suggests that CMG2 plays a vital role in the adhesiveness of pancreatic cancer cells. Enhanced cell–matrix adhesion was not observed in MiaPaCa-2 cells with CMG2 overexpression, suggesting that the adhesive capacity of some pancreatic cancer cells is less dependent on CMG2. On the other hand, MiaPaCa-2 cells are already adhesive compared with other cell lines tested, even with lower expression of CMG2, suggesting that other adhesion molecules are more involved and are sufficient for the adhesion. In the present study, CMG2 promoted the proliferation of PANC-1 cells, but not MiaPaCa-2 and ASPC-1 cell lines, suggesting different roles played by CMG2 in these three cell lines. The reduced proliferation observed in the PANC1 CMG2-knockdown cells can also be at least partially a result of the impaired adhesion. No obvious change in cell proliferation was observed in both MiaPaCa-2 and ASPC-1 cells. CMG2 overexpression also exhibited little effect on the adhesion of MiaPaCa-2 cells, whilst ASPC-1 cells appeared to be more robust even with a loss of adhesion. MiaPaCa2 is the most adhesive cell line in comparison with another two pancreatic cancer cell lines regarding their adhesion to the Matrigel. Its adhesive capacity was barely enhanced by the overexpression of CMG2 suggesting that an alternative adhesion molecule(s) plays a dominating role in this particular cell line, which can represent certain pancreatic tumours that are yet to be fully investigated. On the other hand, the ASPC-1 cell line was originally derived from the ascites of a pancreatic cancer patient, which is representative of pancreatic cancer cells in peritoneal metastasis. It has already acquired certain capability to maintain proliferation with a level of independence from anchorage; meanwhile, this cell line presents a higher level of CMG2 expression, suggesting the involvement of CMG2 in metastasis by coordinating adhesion not just to the ECM, but also to HA and other cells. Similarly, a promotive effect was also previously reported in gastric cancer cells [13], which is in line with the findings in PANC-1 cells. However, our previous studies showed that CMG2 elicited the inhibition of the proliferation of breast cancer cells [9]. Compared with the marked influence of CMG2 on cell adhesion, its impact on migration and invasion in pancreatic cancer cells appeared to be minimal. Taken together, this suggests that CMG2 coordinates cellular functions, including proliferation and migration, through different mechanisms in different cancers and cancer cells depending on the availability of those operational mechanisms, which are yet to be fully elucidated.

The peritoneum is the second-most common metastatic site of pancreatic cancer [25]. During peritoneal metastasis, the adhesion of disseminating cancer cells to the peritoneum is a crucial step before they can invade through the peritoneum. Reduced adhesion to mesothelial cells was found in PANC-1 and ASPC-1 cells with CMG2 knockdown, suggesting that CMG2 plays an important role in the adhesion of pancreatic cancer cells to the peritoneum.

The HA film covering the peritoneum forms a native barrier to prevent the adhesion of disseminating cancer cells. HA-interacting molecules, expressed by cancer cells, are actively involved in peritoneal metastasis [21]. In the present study, it was shown that the CMG2-enhanced adhesion of pancreatic cancer cells to mesothelial cells can be inhibited by treatment with soluble HA. ICAM-1 is one of the HA-interacting molecules [26,27]. An increased expression of ICAM-1 was seen in the pancreatic cancer cells following the overexpression of CMG2. This suggests that in the direct involvement of CMG2 in adhesion, CMG2 can also enhance adhesion through the upregulation of ICAM-1 in pancreatic cancer cells to facilitate the adhesion to HA and mesothelial cells during the dissemination to the peritoneum.

The viability of epithelial cells is maintained by attachment to the ECM. The loss of anchorage can induce apoptosis in epithelial cells, being referred to as anoikis [28,29]. Anoikis is a process that can be initiated by Mcl-1 degradation and Bim induction [30]. In a suspended cell culture, CMG2 promoted survival and prevented apoptosis in the suspended pancreatic cancer cells. The activation of caspase-3 and Bim expression was higher in all three different pancreatic cancer cells with low CMG2 expression. Activated caspase-3 plays an important role in the execution of cell apoptosis [31]. The interaction between EGFR and integrins in colon cancer can promote cell anoikis resistance [32]. Furthermore, CMG2 promoted cell aggregation in MiaPaCa-2 cells. In addition to interacting with HA, with the increased expression of ICAM-1 in MiaPaCa-2^CMG2exp^ cells, ICAM-1 may also contribute to the CMG2-promoted aggregation since it has been shown to be an important adhesion protein mediating cell–cell adhesion [33]. ICAM-1 can promote the formation of CTC (circulating cancer cell) clusters in lung cancer, by promoting cell aggregation [33]. ICAM-1 expression is usually increased in more aggressive cancers, including triple-negative-subtype breast cancer [34] and non-small cell lung carcinoma [35,36]. This suggests that CMG2 can facilitate aggregation/clustering and prevent anoikis in the suspended pancreatic cancer cells, to enhance their survival during peritoneal dissemination.

Focal adhesion complexes play an important role in promoting cell–matrix adhesion and the connection between the ECM and the cytoskeleton, which further promotes cell survival, differentiation, proliferation, and migration [37,38,39]. In addition, cell–ECM adhesion mediated by focal adhesion is associated with higher drug and radiation therapy resistance [40,41,42,43,44]. PTK2 (FAK) is a pivotal molecule in focal adhesion and its downstream signalling. PTK2 can be activated by EGFR [45]. The phosphorylation of PTK2 was highly associated with CMG2 expression in the three pancreatic cancer cells that were examined in the present study. PTK2 interacts with integrins and further promotes cell adhesion [46]. The interaction between integrins and PTK2 can also promote the phosphorylation of PTK2 [47]. Furthermore, integrins, as adhesion molecules to trigger focal-adhesion-related adhesion and motility, can enhance survival and migration [39,48,49,50]. ITGB3 as a member of the integrin family is involved in focal adhesion [51]. In the present study, the upregulation of ITGB3 mediated by CMG2 was also observed in the pancreatic cancer cells. This suggests that CMG2 can also enhance adhesion via the upregulation of ITGB3 and PTK2 (FAK).

In the present study, increased levels of the phosphorylation of EGFR, Shc and ELK1 were revealed in cells with CMG2 overexpression, while reduced phosphorylation was seen in those CMG2-knockdown cell line models, suggesting that the EGFR pathway can be activated by CMG2. Activated EGFR can bind with other ERBB family proteins and further activate downstream signalling, including the RAS–RAF–MEK–ERK–MAPK pathway and PI3K–AKT–mTOR pathways [52,53,54]. EGFR can promote cell proliferation, survival, angiogenesis and metastasis in various cancers [52]. To date, several drugs targeting EGFR and its downstream signalling have been applied to treat cancer, including erlotinib, cetuximab, panitumumab and gefitinib [52]. However, the involvement of EGFR in CMG2-coordinated biological activities in pancreatic cancer cells and corresponding therapeutic opportunities are yet to dissected and evaluated.

Additionally, an EGFR inhibitor, ERK inhibitor and FAK inhibitor can prevent the CMG2-induced expression of ITGB3 and ICAM-1 expression induced by CMG2, which further verified that CMG2 can promote the expression of ICAM-1 and ITGB3 by activating the EGFR and FAK pathway. Taken together, both EGFR and focal adhesion are two important players to be further investigated for their role in the CMG2-regulated cellular functions of pancreatic cancer cells, which can also shed light on their therapeutic potential.

## 5. Conclusions

CMG2 is upregulated in pancreatic cancer. Higher expression of CMG2 in pancreatic cancer is associated with distant metastasis and shorter survival. CMG2 promotes cell–matrix adhesion, cell–cell adhesion, cell–hyaluronic acid adhesion and cell viability during dissemination, in which CMG2 orchestrates EGFR signalling, focal adhesion and HA-interacting molecules are involved. This suggests that CMG2 plays a pivotal role in pancreatic cancer progression and metastasis. However, the impact of the findings can be limited by the samples and cell lines examined in the present study with those techniques employed. Its potential and application for the early diagnosis and target therapy of pancreatic cancer provokes more intensive research by examining metastatic pancreatic tumours, employing patient-derived xenograft, organoid and peritoneal metastatic animal models. CMG2 itself together with its regulation of EGFR, FAK, ITGB3 and ICAM-1 will be further evaluated for their therapeutic potential with precise targeting.

## Figures and Tables

**Table 1:** Transcript levels of CMG2 in pancreatic cancer (Beijing clinical cohort).

Clinical Samples	N	Median (IQR)	*p*-Value
Tumour	153	4 (0~568)	0.002
Normal	175	0 (0–54)	
**Gender**			
Male	93	3 (0~283)	0.5474
Female	60	5 (0~1093)	
**Node status**			
Node negative	60	21 (0~929)	0.4694
Node positive	81	2 (0–157.9)	
**Differentiation**			
High	7	47 (1~15,659)	
Moderate high	13	2 (0~2005)	0.428 vs. High
Moderate	56	2 (0~247)	0.765 vs. High
Moderate low	59	3 (0~584)	0.847 vs. High
low	10	284 (0~5209)	0.24 vs. High
**TNM staging**			
1–2	111	20 (0~1109)	0.483
3–4	24	2 (0~154)	
**T staging**			
1–2	20	11 (0~209)	0.459
3–4	107	19 (0~1554)	
**Clinical outcomes**			
Dead	36	132 (0~1697)	0.093
Alive	108	2 (0~454)	
**Metastasis**			
No	140	6 (0~913)	0.4697
Yes	13	0 (0~107)	

## Data Availability

The sources of public datasets were listed: The Cancer Genome Atlas (TCGA): http://firebrowse.org/, (accessed on 13 October 2019); GSE71729 (accessed on 7 September 2015), GSE15471 (accessed on 13 June 2009) and GSE19650 (accessed on 23 December 2010): https://www.ncbi.nlm.nih.gov/geo/query/acc.cgi (accessed on 5 June 2024); Kmplot: https://kmplot.com/analysis/ (accessed on 4 April 2024); more data are shown in the Supplementary Materials. Further inquiries can be directed to the corresponding author.

## Supplementary Materials

The following supporting information can be downloaded at: https://www.mdpi.com/article/10.3390/cancers16162893/s1, Table S1: Primer sequences. Table S2: Antibodies used for western blot and immunoprecipitation. Figure S1: CMG2 knockdown in PANC-1 cell line. FigureS2: The effect of CMG2 on pancreatic cancer invasion and migration. Figure S3: ICAM-1 and ITGB3 expression in PANC-1 cell line treated with inhibitors. Figures S4 and S5: All the original figures of western blot.
