# Supplementary material for: Implication of Capillary Morphogenesis Gene 2 (CMG2) in the Disease Progression and Peritoneal Metastasis of Pancreatic Cancer
Source: Cancers (Basel). 2024 Aug 20;16(16):2893. doi: 10.3390/cancers16162893 (PMC11352480; doi:10.3390/cancers16162893)
Supplement: Supplementary file 1 [file cancers-16-02893-s001.zip › cancers-3061895-supplementary.pdf]

Supplementary Table S1. Primer sequences

Genes	Primer name	Sequence (5'-3')	Amplicon size (bps)	Reference sequence
GAPDH	Forward	GGCTGCTTTTAACTCTGGTA	421	NM_001357943.2
	Reverse	GACTGTGGTCATGAGTCCTT		
CMG2	Forward	CAAAATCAGTAAAGGCTTGG	805	NM_001145794.2
	Reverse	CAAAGGTTCTTCTTCCTCCT		
GAPDH	Forward	CTGAGTACGTCGTGGAGTC	92	NM_001357943.2
	Reverse	*ACTGAACCTGACCGTACA*CAGAGATGATGA CCCTTTTG		
CMG2	Forward	AGCCTTTGATCTCTACTTCG	87	NM_001357943.2
	Reverse	*ACTGAACCTGACCGTACA*GCAAGTTGCTGT ACGAAATTA		
ICAM-1	Forward	ACAGCTAAAACCTTCCTCAC	105	NM_000201.3
	Reverse	*ACTGAACCTGACCGTACA*CTGGC AGCGTAGGGTAAG		
ITGB3	Forward	CAGATTTGCCTTATTGGCAG	117	NM_000212.3
	Reverse	*ACTGAACCTGACCGTACA*AAATG TCTACAGCAGTGAGG		
CMG2 ribozyme	Forward	CTGCAGAATAAACACTAGCCCCAAGTCTGA TGAGTCCGTGAGGA		
	Reverse	ACTAGTAGAGGCAAAGATATCCAGGTTTCG TCCTCACGGACT		

Note: Reverse primers with a sequence in italic (known as Z-sequence) were used for the QPCR.

Supplementary Table S2. Antibodies used for western blot and immunoprecipitation

Protein	Product code	Species	Supplier	Molecular weight (kDa)
GAPDH	SC 32233	Mouse	Santa Cruz Biotechnology Ltd	37 kDa
ANTXR2	16723-1-ap	Rabbit	Protein tech Ltd	54 kDa
Actin	SC1615	Goat	Santa Cruz Biotechnology Ltd	43 KDa
EGFR	SC 71034	Mouse	Santa Cruz Biotechnology Ltd	170 kDa
ICAM-1	SC 8439	Mouse	Santa Cruz Biotechnology Ltd	85~110 kDa
ICAM-1	SC 8439	Mouse	Santa Cruz Biotechnology Ltd	85-110 kDa
FAK	SC 1688	Mouse	Santa Cruz Biotechnology Ltd	125 kDa
ELK-1	SC 365876	Mouse	Santa Cruz Biotechnology Ltd	62 kDa
Shc	SC 967	Mouse	Santa Cruz Biotechnology Ltd	46-66 kDa
Bim	SC11425	Rabbit	Santa Cruz Biotechnology Ltd	19-24kDa
BCL-1	SC 509	Mouse	Santa Cruz Biotechnology Ltd	26 kDa
Caspase3	SC 7148	Rabbit	Santa Cruz Biotechnology Ltd	32, 11, 17, 20 kDa
p-Tyr	SC 508	Mouse	Santa Cruz Biotechnology Ltd	
p-Thr	SC 5267	Mouse	Santa Cruz Biotechnology Ltd	
P-Ser	SC 81517	Mouse	Santa Cruz Biotechnology Ltd	
**Secondary antibody**	**Type**	**Product Code**	**Supplier**	
Mouse	IgG	A-9044	Sigma-Aldrich Ltd	
Rabbit	IgG	A-9169	Sigma-Aldrich Ltd	
Goat	IgG	A-5420	Sigma-Aldrich Ltd	

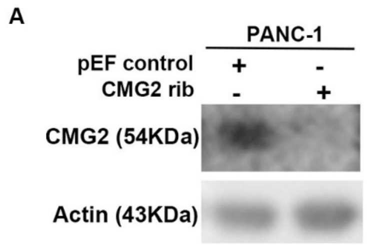

Supplementary Figure S1: CMG2 knockdown in PANC-1 cell line. Western blot results show that the CMG2 expression was knocked down in the PANC-1 cell line using ribozyme.

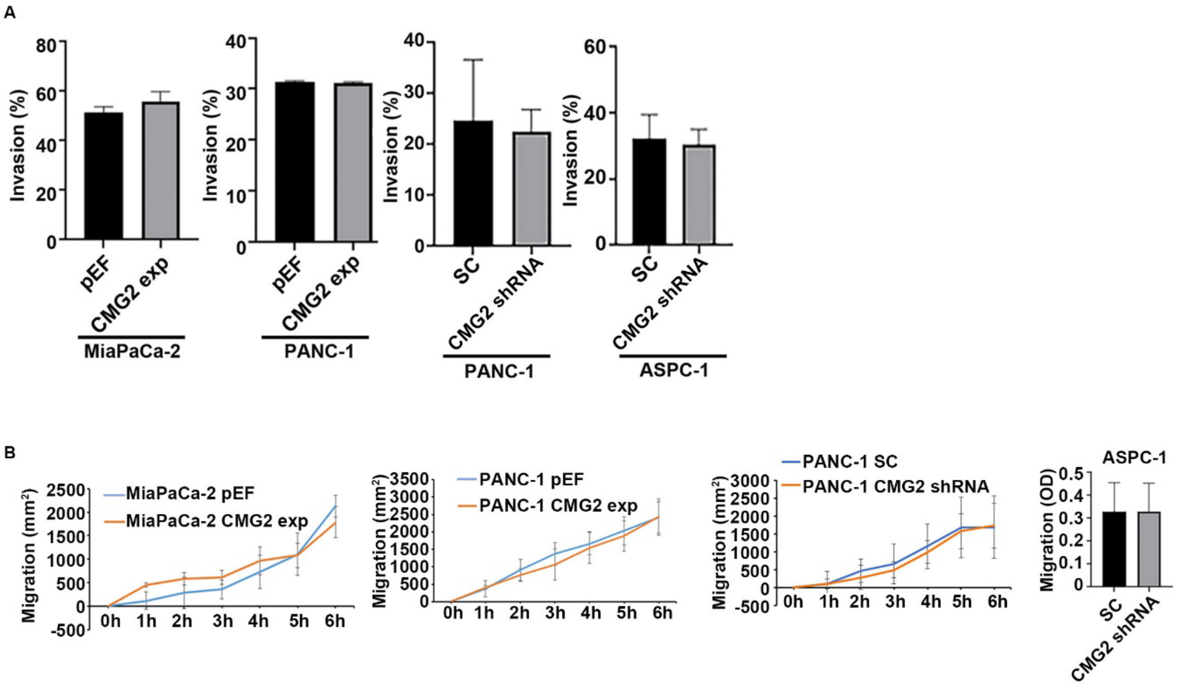

Supplementary Figure S2: The effect of CMG2 on pancreatic cancer invasion and migration. (A) The influence of CMG2 on the invasion of MiaPaCa-2, PANC-1 and ASPC-1 was determined using Matrigel. Six replicates were examined for each cell line in an experiment. (B) The influence of CMG2 on cell migration was checked by wound healing test in MiaPaCa-2 and PANC-1 cell lines. Microcarrier beads were used for the ASPC-1 cell line.

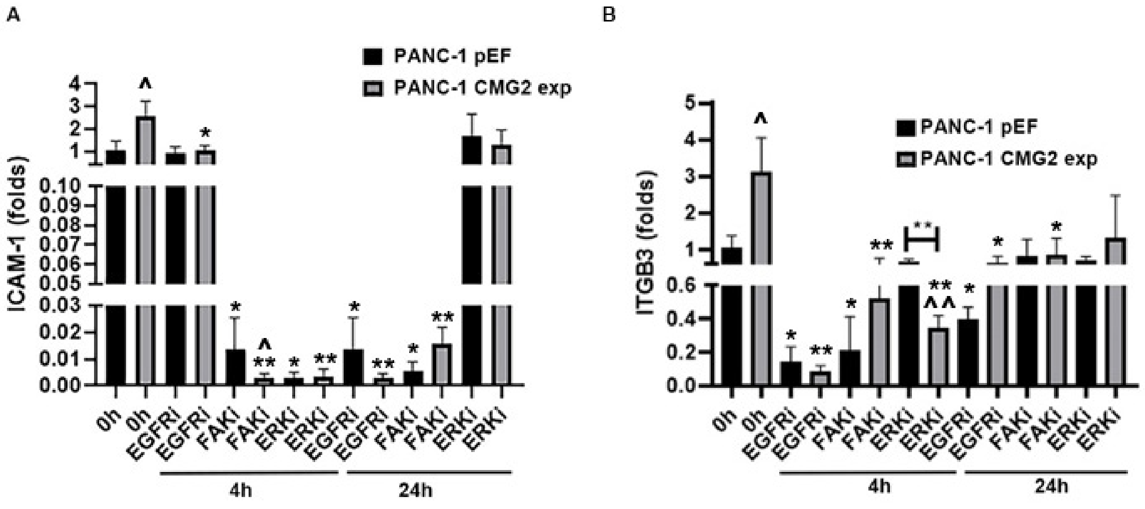

Supplementary Figure S3:QPCR was done to check the expression of ICAM-1 (A) and ITGB3 (B) in the PANC-1 cell line with CMG2 overexpression, which was treated by EGFR inhibitor (Gefitinib, 400nM), FAK inhibitor 14 (400nM) and ERK inhibitor (FR18024, 200nM). Cell lines were treated with small inhibitors for 4 and 24 hours. * v.s. untreated control, ^ v.s. pEF corresponding control. ***p<0.001, **p<0.01, *p<0.05, ^^^p<0.001, ^^p<0.01, ^p<0.05.

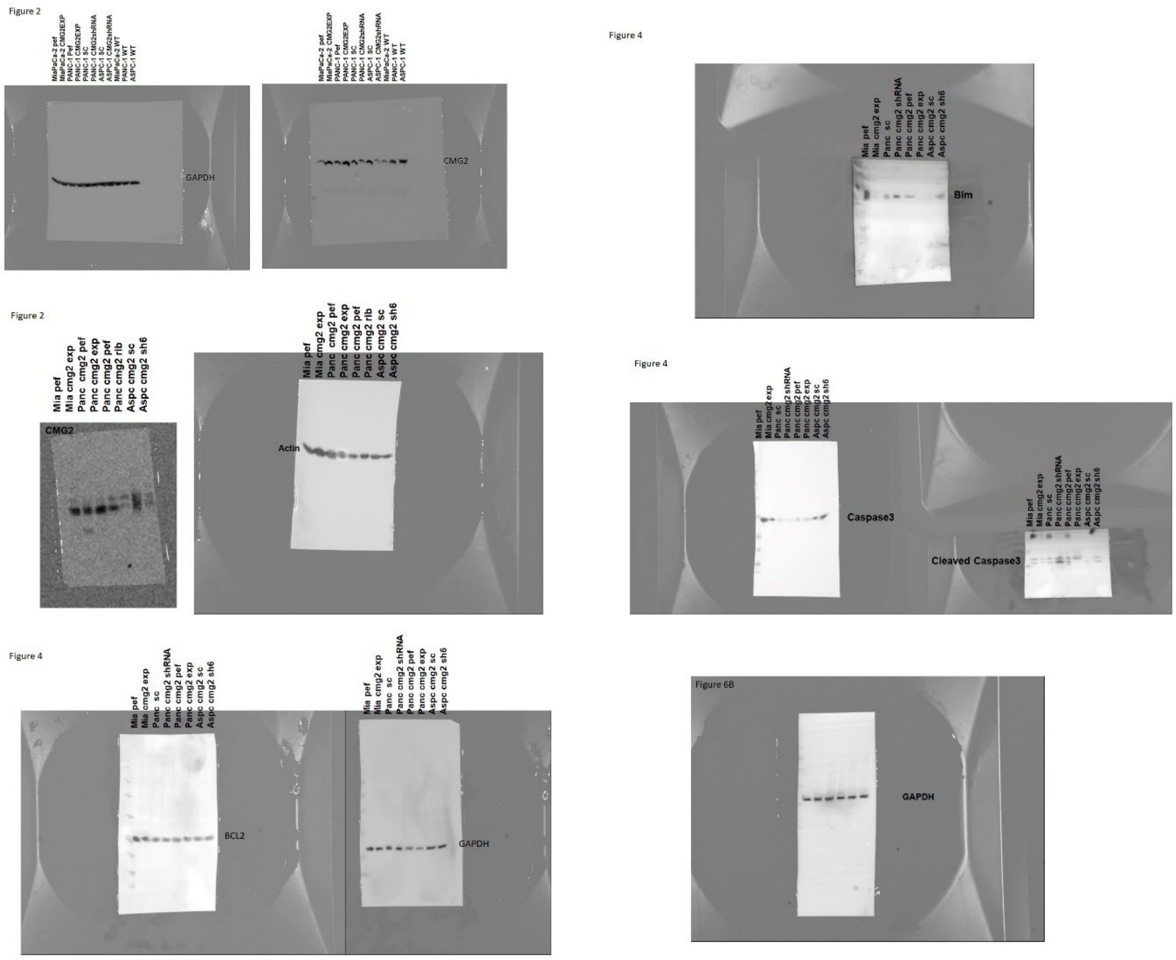

Supplementary Figure S4: Original images of western blots (1).

Figure 6

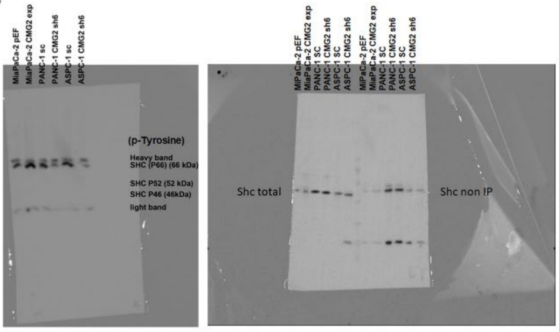

Figure 6

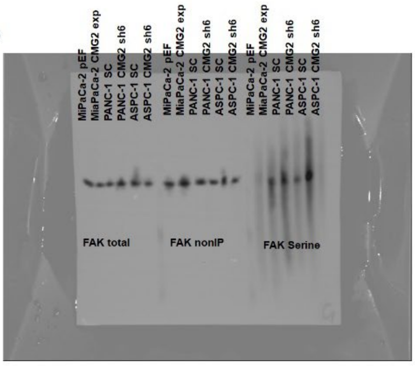

Figure 6

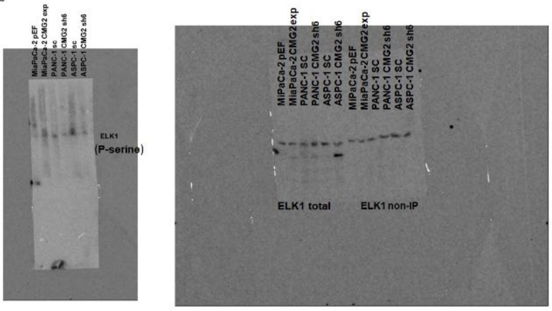

Figure 6

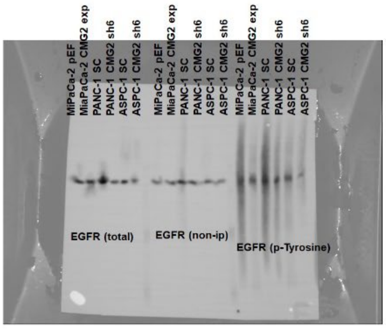

Figure 7

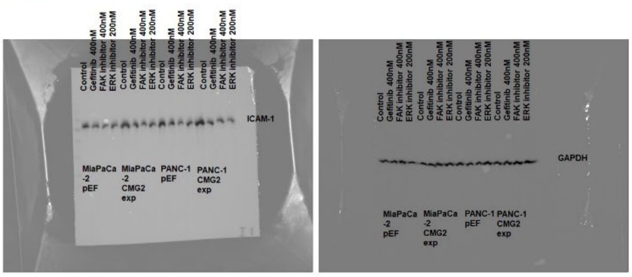

Supplementary figure S5: Original images of western blots (2).
